# Evaluating the safety and cardiac impact of resistance training in anthracycline-treated patients: a systematic review

**DOI:** 10.1186/s40959-025-00425-3

**Published:** 2026-01-12

**Authors:** Johnny Huang, Rex Lam, Alice Pozza, Hadeel Hassan, Jane E. Schneiderman, Paul C. Nathan, Luc Mertens, Barbara Cifra

**Affiliations:** 1 Division of Respiratory Medicine, The Hospital for Sick Children, Toronto, Canada; 2 Faculty of Health, School of Kinesiology & Health Science, York University, Toronto, Canada; 3 Division of Cardiology, The Labatt Family Heart Centre, The Hospital for Sick Children, University of Toronto, Toronto, Canada; 4 Division of Haematology/Oncology, The Hospital for Sick Children, University of Toronto, Toronto, Canada; 5 Faculty of Kinesiology & Physical Education, University of Toronto, Toronto, ON Canada

**Keywords:** Anthracycline, Aerobic training, Resistance training, Cardiotoxicity, Cancer survivors

## Abstract

Anthracycline chemotherapy is commonly used to treat cancer in both adult and pediatric patients. While effective, anthracycline treatment is associated with a high risk of cardiotoxicity, often manifesting as a decline in left ventricular function, resulting in long term cardiovascular complications. Aerobic exercise has been studied extensively for its cardioprotective benefits in patients with anthracycline-induced cardiac dysfunction; however, the role of resistance training remains unexplored and controversial due to historical concerns regarding safety. This systematic review examined the existing literature on the effects and safety of resistance training in pediatric and adult cancer patients treated with anthracyclines. A two-part screening process was completed in Covidence (2025), starting with the screening of titles and abstracts, followed by the full-text screening. Eight studies, all incorporating AR-T were reviewed. The findings suggest that AR-T is safe and well-tolerated, with no evidence indicating that resistance training exacerbates cardiac dysfunction. No studies included exclusively pediatric or adolescent patients, limiting the generalizability of the findings to these populations. Randomized controlled trials focused solely on resistance training are needed to inform future clinical guidelines.

## Introduction

Cancer in children and adolescents is rare but remains a leading cause of disease-related death in the United States, with an estimated 14,910 diagnoses and 1590 deaths in 2024 [1]. Despite this, 5-year survival rates have improved significantly from 58% in children (ages 0–14) and 68% in adolescents (15–19) in the 1970s to over 80% across age groups between 2013 – and 2019 [1]. As survivorship increases, treatment-related side effects have become a growing clinical concern [2].

Anthracyclines are widely used chemotherapeutic agents that inhibit DNA and RNA synthesis and are associated with dose-dependent cardiotoxicity [3, 4]. This cardiotoxicity can lead to cardiac dysfunction, with mechanisms believed to be multifactorial and progressive [5]. Patients exposed to anthracyclines require long-term cardiovascular monitoring [5]. Cardiotoxicity may present acutely (during or soon after treatment), early (within the first-year post-treatment), or late (beyond one year) [3]. Cumulative doxorubicin ≥ 550 mg/m^2^ are linked to a 7.2% 30-year incidence of heart failure, seven times higher than in pediatric cancer survivors who did not receive anthracycline [6–8]. Echocardiography is the primary tool for cardiac monitoring, aimed at detecting reversible subclinical dysfunction [9]. To standardize diagnosis and care, the term cancer therapy-related cardiac dysfunction (CTRCD) has been proposed to describe heart complications stemming from treatment [2].

With cardiotoxicity contributing to long-term morbidity and mortality, there is increasing interest in preventative strategies. Exercise has emerged as a potential intervention. Aerobic and resistance training (AR-T) are associated with improved physical function, quality of life, and possibly a cardioprotective effect via redox regulation and cell survival mechanisms [5, 6, 10, 11], however these benefits remain incompletely understood in the context of anthracycline exposure.

Resistance training, shown to improve strength and muscular endurance, is rarely recommended to cancer patients treated with anthracyclines due to concerns that the increased blood pressure during training may elevate cardiac wall stress and afterload, potentially exacerbating myocardial damage [5, 12–17]. As a result, many survivors are discouraged from participating in resistance training [18].

Research on isolated resistance training in this population remains limited as most studies focus on isolated aerobic training or AR-T programs. There are no specific, evidence-based guidelines for implementing resistance training during or after anthracycline treatment, and recommendations vary among clinicians [5, 13]. As survival rates improve, cardiovascular disease (CVD) driven by aging and cancer treatment-related side effects, has become a major cause of long-term morbidity and mortality. This highlights the need for structured, exercise-based interventions, potentially modelled after cardiac rehabilitation, to reduce CVD risk in survivors [19]. This systematic review aims to examine the current literature on the safety and effects of resistance training in adult and pediatric cancer patients who are receiving or have received anthracyclines.

## Methods

### Study selection

Studies eligible for inclusion were experimental human studies published after 2014, that investigated the effects of resistance training or a combination of aerobic and resistance training on cardiac function, specifically left ventricular dysfunction, in adults, teens, and/or children who had received or were currently receiving anthracycline-based chemotherapy.

### Search strategy

The search was conducted in Ovid MEDLINE(R) and Epub Ahead of Print, In-Process, In-Data-Review & Other Non-Indexed Citations and Daily < 1946 to February 5, 2024>; University of Sydney Physiotherapy Evidence Database (PEDro); and Ebscohost SPORTDiscus (April 8, 2024). The search strategy consisted of both controlled vocabularies, such as the National Library of Medicine’s MeSH (Medical Subject Headings) terms and SPORTDiscus thesaurus terms, and keywords. Results were limited to English language publications, between 2014 and 2024.

### Data synthesis & analysis

**Fig. 1:**
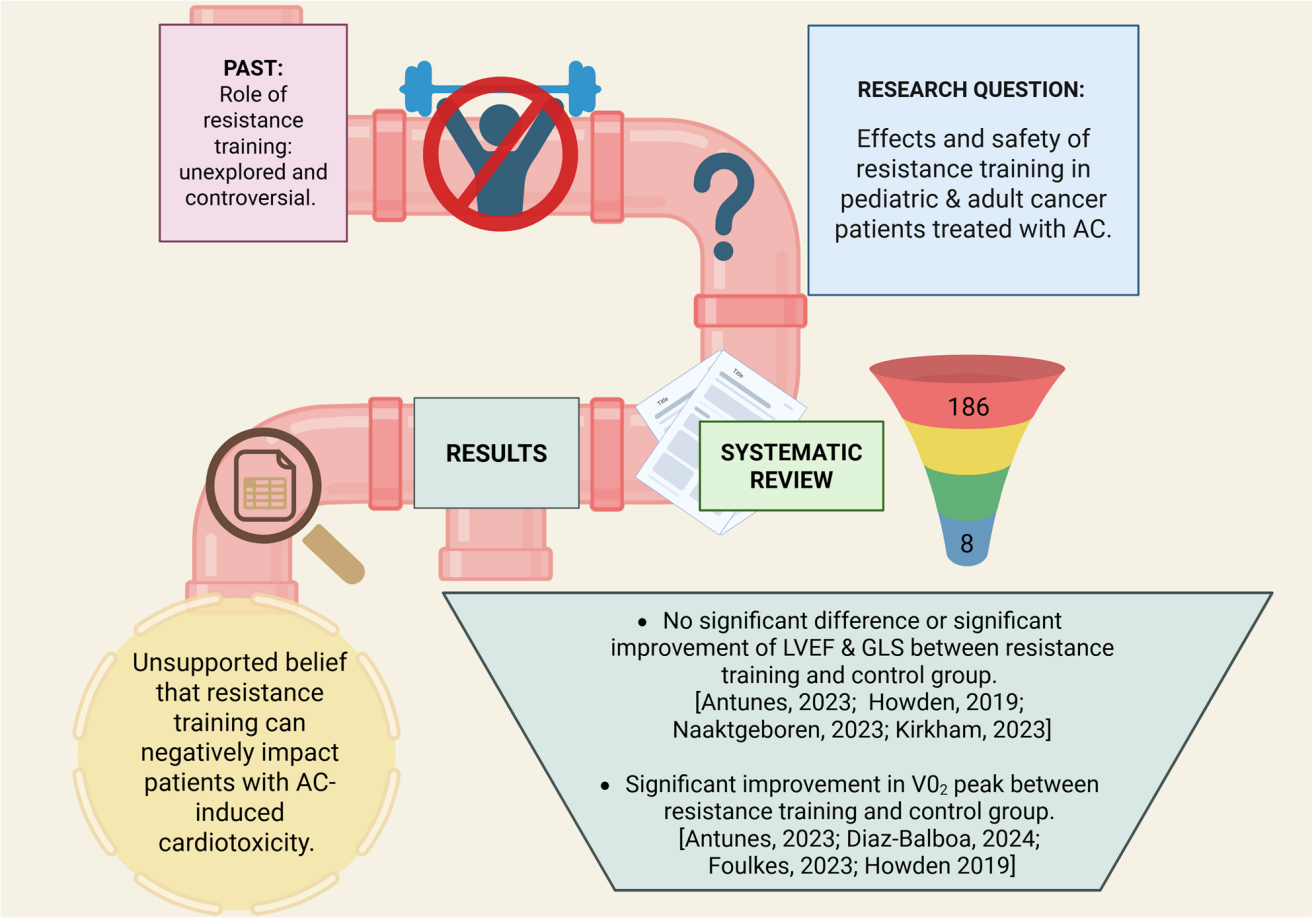
Summary of the systematic review process and key findings. AC: anthracycline; LVEF: left ventricular ejection fraction; GLS: global longitudinal strain; VO₂: peak oxygen consumption

A two-part screening process was completed in Covidence (2025) by two authors separately (R.L., & J.H.), starting with the screening of titles and abstracts, followed by the full-text screening. Conflicts in accepting and excluding articles were then reviewed together to ensure the article matched the inclusion criteria. Data extracted from eligible studies included author and year of publication, study design/intervention protocol, population, and results (Fig. 1).

## Results

**Fig. 2:**
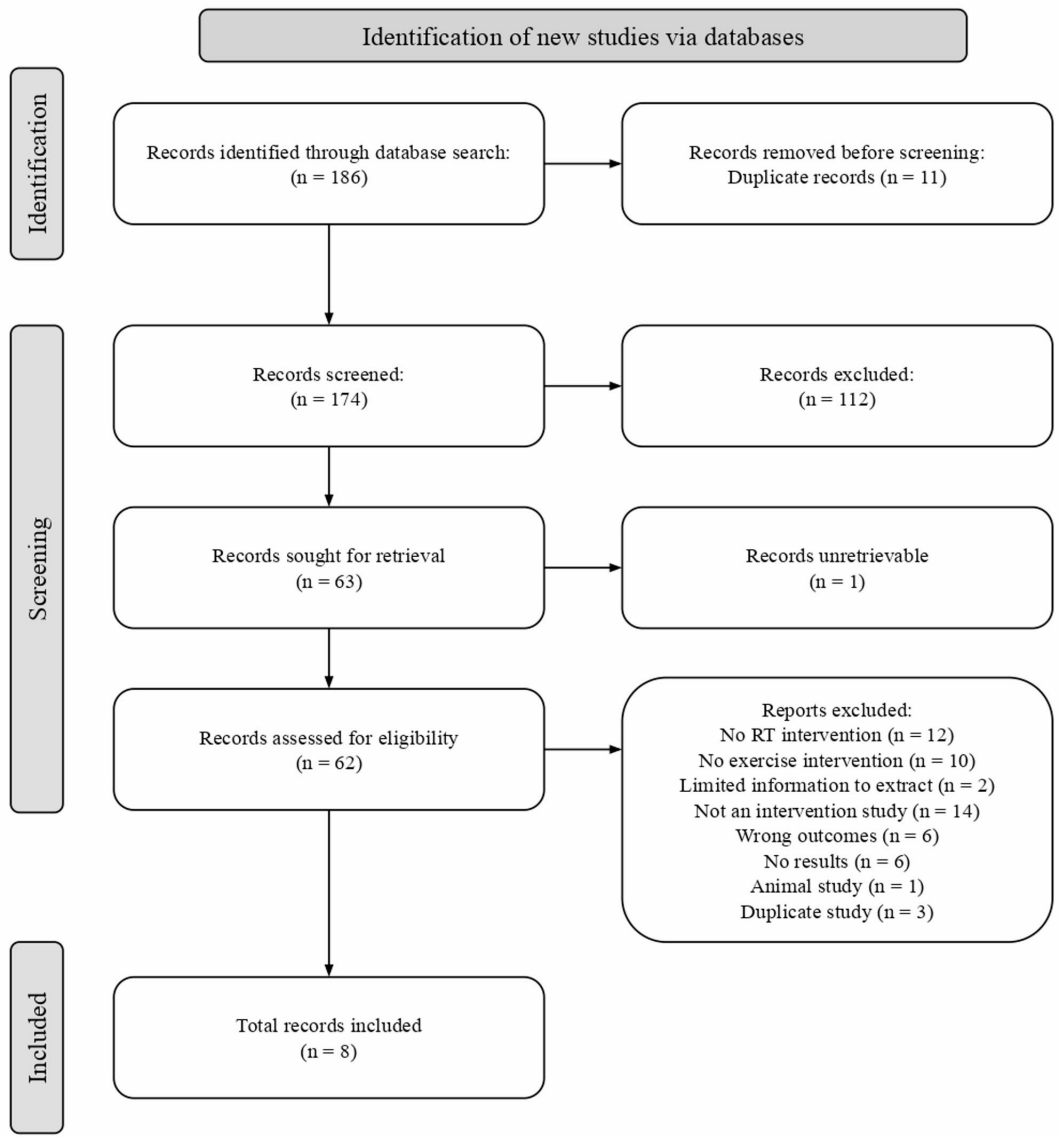
PRISMA flow diagram of included studies

Using the search strategy described above, 186 studies were identified, 11 of which were duplicates. 174 studies were screened based on their titles and abstracts, with 112 found to be irrelevant. Full-text screening was completed on 63 studies with 54 studies deemed ineligible. 8 studies were included in the systematic review (Fig. 2). A summary of the characteristics of the included studies can be found in Table 1.

**Table 1:** Characteristics of included studies

Study ID	Design	Title	Aim	Criteria
Antunes 2023 [14]	Randomized controlled trial	Effects of exercise training on cardiac toxicity markers in women with breast cancer undergoing chemotherapy with Anthracycline: a randomized controlled trial	To determine the effects of a supervised AR-T program on cardiotoxicity markers and cardiorespiratory fitness in women with early-stage BC receiving anthracycline-containing chemotherapy	Women (≥ 18 years of age), early-stage BC, scheduled to receive anthracycline. Exclusion criteria: contraindication to maximal exercise testing; diabetes mellitus; severe anemia (hemoglobin < 8 g/dL); history of CVD; taking beta-blockers.
Diaz-Balboa 2024 [15, 16]	Randomized controlled trial	Exercise-based cardio-oncology rehabilitation for cardiotoxicity prevention during breast cancer chemotherapy: The ONCORE randomized controlled trial	To determine the effectiveness of an exercise-based CORe program in preventing CTRCD [left ventricular ejection fraction (LVEF) drop ≥ 10% to a value < 53% or a decrease > 15% in GLS]. Secondary outcomes were changes in cardiac biomarkers, physical performance including peak oxygen consumption, psychometric and lifestyle outcomes. Safety, adherence, and patient satisfaction were also assessed.	Women (18–70 years), first diagnosis of stage I-III BC, scheduled to receive adjuvant/neoadjuvant anthracycline and/or anti-HER2 antibodies, without contraindications to an exercise-based CORe program. Exclusion criteria: previous CVD and ≥ 20% of the planned cardiotoxic chemotherapy at enrollment.
Foulkes 2023 [17, 19]	Randomized controlled trial	Exercise for the Prevention of Anthracycline-Induced Functional Disability and Cardiac Dysfunction: The BREXIT Study	The primary aims of this study were to: (1) determine if 12-months of AR-T commenced at the onset of anthracycline treatment can reduce the proportion of BC patients with functional disability (peak VO_2_, < 18 ml/kg/min), and (2) compare current standard-of-care for detecting cardiac dysfunction (resting left-ventricular ejection fraction assessed from 3-dimensional echocardiography) to measures of cardiac reserve (peak exercise cardiac output assessed from exercise CMR) for predicting the development of functional disability 12 months following anthracycline treatment. Secondary aims were to assess the effects of AR-T on VO_2_peak, left ventricular morphology, vascular stiffness, cardiac biomarkers, body composition, bone mineral density, muscle strength, physical function, habitual PA, cognitive function, and multidimensional quality of life.	Females, 40 to 75 years, stage I to III BC, scheduled for anthracycline treatment. Exclusion criteria: history of structural heart disease; contraindication to CMR; absolute contraindication to exercise according to the American College of Sports Medicine; non–English Speaking; significant cognitive impairment.
Howden 2019 [20]	Prospective, non-randomized controlled trial	Exercise as a diagnostic and therapeutic tool for the prevention of CV dysfunction in BC patients	To determine (a) whether a structured AR-T program would attenuate reductions in VO_2_peak and (b) whether exercise cardiac imaging is a more sensitive marker of cardiac injury than the current standard of care resting left ventricular ejection fraction (LVEF).	Women with early-stage BC recruited from local hospitals in the Melbourne Metropolitan area. Exclusion criteria: known structural heart disease, arrhythmias, or contraindication to CMR.
Kirkham 2020 [21]	Prospective, non-randomized controlled trial	Exercise training affects hemodynamics not cardiac function during Anthracycline-based chemotherapy	To assess whether AR-T during anthracycline-based chemotherapy for treatment of BC affects resting cardiac function and hemodynamics.	Women diagnosed with stage I-III BC, scheduled to receive anthracycline treatment. Exclusion criteria: history of CVD and conditions associated with CV risk; BMI > 35 kg/m2, current smoker status, uncontrolled hypertension, or uncontrolled diabetes: participants enrolled in a concurrent pharmacological cardio-protection study.
Naaktgeboren 2023 [10]	Long-term follow-up of two previously performed prospective RCTs; the PACT and PACES studies conducted between 2009–2013	Effects of exercise during chemotherapy for breast cancer on long-term CV toxicity	To evaluate the effects of an AR-T intervention during adjuvant chemotherapy for BC on mitigating long-term cardiotoxicity and impaired cardiorespiratory fitness compared with non-exercise controls	All BC participants of the original PACT and PACES study: included participants with non-metastatic BC, free or serious comorbidities (e.g., CVD) that would impede PA. Exclusion Criteria: patients who died during follow-up; considered ineligible by their treating physician; received chemotherapy, targeted therapy or thoracic irradiation for recurrent or metastasis BC or other malignancies after completion of the original PACT and PACES; those who dropped out during the original PACT study or actively declined invitations for future follow-up at the time of the first follow-up.
Kirkham 2023 [13]	Randomized controlled trial	TITAN Trial: A Randomized Controlled Trial of a Cardiac Rehabilitation Care Model in Breast Cancer	To evaluate the effectiveness of a multidisciplinary model of cardiac rehabilitation to reduce cardiotoxicity and improve CV risk in patients undergoing BC treatment.	Women, age > 18 years, early stage I-III BC scheduled to receive anthracycline and/or trastuzumab-based chemotherapy and English-speaking. Exclusion criteria: contraindications to MRI/exercise testing, previous heart failure, baseline LVEF < 50%, or prior cardiotoxic treatment.
Schneider 2023 [9]	Randomized controlled trial	Supervised exercise training in patients with cancer during anthracycline-based chemotherapy to mitigate cardiotoxicity: a randomized-controlled-trial	To compare changes in markers of cardiac function and myocardial injury between patients performing supervised AR-T during anthracycline-based chemotherapy and patients receiving only PA advice.	Women and men aged ≥ 18 years with historically confirmed BC and lymphoma who were scheduled for first time anthracycline treatment. Exclusion criteria: known or newly diagnosed structural heart disease; contraindication to maximal cardiopulmonary exercise testing (CPET); cancer-specific contraindications to exercise; previous radiotherapy of mediastinum and/or the left breast; significant cognitive impairment or inability to follow study procedures (i.e., language problems)

*Abbreviations*: *BC* Breast Cancer, *CMR* Cardiac Magnetic Resonance Imaging, *CPET* Cardiopulmonary Exercise Test, *CV* Cardiovascular, *CVD* Cardiovascular Disease, *TRCD* Cancer Therapy-related Cardiac Dysfunction, *GLS* Global Longitudinal Strain, *HF* Heart Failure, *LVEF* Left Ventricular Ejection Fraction, *PA* Physical Activity

All 8 studies included investigated the use of a combined AR-T protocol for patients who had received, were currently receiving, or were scheduled to receive anthracyclines. No studies investigated the use of resistance training alone as an intervention. Detailed exercise protocols can be found in Table 2. All studies featured adult participants (≥ 18 years). All studies included females with early-stage breast cancer (BC), with one study including both females with BC and males with lymphoma [9]. Participant characteristics for each study are summarized in Table 3. Outcomes investigated in the included studies included left ventricular ejection fraction (LVEF), global longitudinal strain (GLS), peak oxygen uptake (VO_2_peak), and other cardiac biomarkers. Significant findings from each study, are summarized in Table 4.

**Table 2:** Exercise intervention characteristics for cancer patients who have received or are receiving anthracycline therapy

Study ID	Mode	Frequency (Sessions/week)	Intensity	Time	Type	Duration	Instructor	Exercise Session Adherence (%)
Antunes 2023 [14]	Combined aerobic and resistance training	3	*Aerobic*: Week 1–2 < 50% HRR. Week 3 onward 65–80% HRR. *Resistance*: Week 1–2, lowest available load or no resistance 2 × 10 reps. After, resistance was added until patients could perform 3 × 12RM. When patients were able more than 12 RM for 3 sets at the set weight for 3 consecutive sessions, an increased load of 5–10% was considered.	5–10 min warm-up *Aerobic*: Start with 20 min. 3 min increase per 2 weeks for a total duration of 30 min. *Resistance*: Not specified. 5 min cooldown with stretching exercises.	*Aerobic*: Treadmill, stationary bike, or stepping. *Resistance*: Upper body (shoulder press, chest press, lateral pulldown, biceps curls, and triceps extension) and lower body weight training exercises (squat, calf raise, leg press, leg extension, and leg curl).	20–24 weeks	Exercise physiologist and physiotherapist	Mean adherence of 63.2 ± 26.9% of the planned exercise sessions. *Aerobic training*: median adherence to duration was 100% [98–100%], and mean adherence to aerobic intensity was 77.9 ± 14.3%. Mean adherence to resistance training prescription (considering prescribed load, sets, and repetitions) was 74.5 ± 11.5%.
Diaz-Balboa 2024 [15, 16]	Combined aerobic and resistance training	2	*Aerobic*: 50–85% HRR *Resistance*: Not stated.	60 min	*Usual Care*: PA advice via phone every 2 months.	3–12 months	Physiotherapist	Overall adherence was 67%. 12 of 54 patients (23%) attended > 80% of the prescribed sessions; 26 patients (48%) attended between 60 and 80%, and 15 patients (28%) attended < 60%. 12 HER2 + patients underwent BC surgery during the program and were unable to attend the sessions on the postoperative days, which were recorded as medical absences.
					*CORe Group*: Body weight and elastic band exercises + treadmill, elliptical, or bicycle.			
Foulkes 2023 [17, 19]	Combined aerobic and resistance training	3–4	**Phase 1**: *Continuous aerobic training*: 10–20 bpm below %HRR at VT measured during baseline CPET. Progressive increases in exercise intensity (i.e., HRR closer to %HRR achieved during VT) each week. *Interval aerobic training*: Progress from %HRR achieved at VT, to 85–95% HRpeak *Resistance training*: 1–2 sets of 8–15 reps 60–70% 1RM Progress to 70–85%1RM 1–2 min rest between sets Intensity was reduced by ~ 5% during the treatment weeks. **Phase 2**: Intensity remains the same as the end of phase 1. 10% reduction in aerobic exercise intensity and reduction to 1 set of each resistance exercise during week 5 and 10 of phase 2 to facilitate recovery.	5 min warmup and cooldown. *Aerobic*: Continuous training: 30–60 min Interval training: 4 × 2–4 min (3 min light cycling between intervals) *Resistance*: unspecified	*Aerobic*: Interval training: cycle ergometer. Continuous training: upright cycle, treadmill, and/or elliptical trainer. *Resistance*: 6 compound exercises (3 upper & 3 lower body). Examples of the exercises to be incorporated in the program include leg press, squats, lunges, step-ups, chest press, overhead press, seated row, and latissimus dorsi pulldown. Exercises were changed or slightly modified to provide training variety and progression.	Phase1: 12 weeks Phase 2: 14 weeks Phase 3: 26 weeks	Exercise physiologist Phase 1: supervised Phase 2: semi supervised Phase 3: unsupervised	Median exercise adherence over 12 months was 73%. Adherence in phase 1, 2, and 3 was 83%, 73%, and 70%, respectively.
Howden 2019 [20]	Combined aerobic and resistance training	2 supervised, 1 unsupervised	Based on baseline maximal exercise test and regular submaximal incremental exercise tests performed through the intervention. 2:1 step paradigm (2 weeks of loading, 1-week deloading)	*Supervised*: 30 min aerobic 30 min resistance *Unsupervised*: 30–60 min aerobic	*Supervised*: Aerobic and resistance training *Unsupervised*: Home-based aerobic exercise session	8–12 weeks	Exercise physiologist	Half of the participants completed 80% of the 24 prescribed sessions (group average 76%, range 38–88%).
Kirkham 2020 [21]	Combined aerobic and resistance training	3	*Aerobic*: 50–75% age-predicted HRR with progressions every 1–2 weeks as tolerated. *Resistance*: moderate intensity	*Aerobic*: 20–30 min *Resistance*: Not stated	*Aerobic*: Treadmill, elliptical, or cycle ergometer *Resistance*: Whole-body	Not stated	Not stated	EX group attended a median of 63% (range 0–96%) of available 3x/wk (supervised. Adherence to prescribed aerobic intensity and duration were 86% and 96%, respectively.
Naaktgeboren 2023 [10–12]	Combined aerobic and resistance training	**PACT**: 2 supervised Encouraged to be physically active at least 3 days **PACES**: All groups encouraged to engage in PA 5 day/week OnTrack: additional 2 supervised	**PACT**: Aerobic: Alternating HR at and below VT measured during baseline testing. Resistance: 2 × 10 at 65% 1RM gradually increasing to 1 × 10 at 75% 1RM and 1 × 20 at 45% 1RM by the end of the program. **PACES**: Onco-Move: 12–14 RPE (Borg scale) OnTrack: Resistance − 2 × 8 at 80% 1RM Aerobic − 50–80% of max workload estimated by the Steep Ramp Test. RPE 12–16 (Borg scale).	**PACT**: 60 min total including warm-up (5 min), aerobic and muscle strength training (25 min each), and a cool-down (5 min). Aerobic: interval at VT (3 × 2 min increasing to 2 × 7 min). Interval below VT (3 × 4 min decreasing to 1 × 7 min) Unsupervised: at least 30 min **PACES**: Onco-Move: at least 30 min OnTrack: 20 min resistance training, 30 min aerobic exercise	**PACT**: Aerobic: interval training Resistance: all major muscle groups (arms, legs, shoulder, and trunk) **PACES**: Onco-Move: PA, no specifics stated. OnTrack: Resistance − 6 large muscle groups. No specifics stated. Aerobic - not stated	**PACT**: 18 weeks **PACES**: Started with the first cycle of chemotherapy and Ended 3 weeks after the last cycle.	**PACT**: Physiotherapist **PACES**: OnTrack: Physiotherapist	**PACT**: Patients participated in 83% (interquartile range, 69%–91%) of the classes offered. **PACES**: OnTrack: attended 71% of planned sessions. Exercise diary: 48% of the OnTrack group and 55% of the Onco-Move group followed the recommendations regarding daily activity levels at least 75% of the time.
Kirkham 2023 [13]	Combined aerobic and resistance training	2 supervised Recommend 1–2 days at home	*Aerobic*: 60–90% HRmax or RPE 3–8 (0–10 Borg scale) *Resistance*: 2 × 10–15 RPE 3–8 (0–10 Borg scale) Progression implemented approximately every 4 weeks, as tolerated. *Home*: Not prescribed	Total: 60–90 min *Aerobic*: 10–60 min *Resistance*: not stated *Home*: not prescribed	*Aerobic*: Cycle ergometer, elliptical or treadmill *Resistance*: Eight whole-body exercises (seated row, bench press, latissimus dorsi pulldown, shoulder press, arm curl, triceps extension, leg press, leg extension and leg curl), and two core strength exercises. *Home*: Aerobic exercise (walking cycling)	52 weeks	Exercise physiologist	Not Stated
Schneider 2023 [9]	Combined aerobic and resistance training	*Pre COVID-19*: 2 supervised, 1 unsupervised *During COVID-19*: 1 supervised, 2 unsupervised	*Aerobic*: Workload corresponding with the patient’s first VT measured during baseline CPET. Progress to 13 on the Borg scale. *Resistance*: 70–80% of 1RM, 2–3 sets of 8–12 reps, 15 on the Borg scale.	*Pre COVID-19*: 90 min total 5 min warm-up 3 min cool-down Aerobic: 30–40 min Resistance: 40–45 min *During COVID-19*: 60 min resistance training only	*Aerobic*: Cycle ergometer *Resistance*: All major muscle groups, coordination and/or balance training. No specific exercises stated.	12 weeks	Exercise therapist	*Presented as median [1st & 3rd quartile]* Centre-based adherence was 65% [36, 78] in the EXduringAC group and 71% [38, 88] in the EXpostAC group. Total adherence (centre- and home-based) for the EXduringAC and the EXpostAC groups were 71% [59, 93] and 87% [67, 100], respectively.

Information provided in Naaktgeboren 2023 is from 2 previously completed studies: the Physical Activity during Cancer Treatment [PACT] study and the Physical Exercise during Adjuvant Chemotherapy Effectiveness Study [PACES], as Naaktgeboren 2023 is a follow-up study to them

*Abbreviations*: *1RM* one repetition maximum, *CPET* Cardiopulmonary exercise test, *EXduringAC* Exercise during anthracycline treatment, *EXpostAC* Exercise post anthracycline treatment, *HRpeak* Peak heart rate, *HRR* Heart rate reserve, *min* minutes, *PA* Physical activity, *RPE* Rate of perceived exertion, *VT* Ventilatory threshold, *wk* week

**Table 3:** Cancer participant characteristics

Study ID	Group	Number of Participants	Age (years)	BMI (kg/m^2^)	Study Attrition
Antunes 2023 [14]	Control	F = 46	51.02 ± 9.54	28.69 ± 6.82	N/A
	Exercise	F = 47	49.66 ± 9.43	26.94 ± 4.32	3 did not start the exercise intervention, 12 discontinued the exercise intervention, and 32 remained enrolled during the whole period of chemotherapy.
Diaz-Balboa 2024 [16]	Control	F = 62	48.92 ± 8.51	48.82 ± 8.02	4 lost to follow-up due to worsening oncological prognosis (*n* = 3) and an ankle fracture (*n* = 1). 58 completed the final assessment.
	Exercise	F = 60	26.86 ± 5.31	26.36 ± 5.77	6 dropped out of intervention due to family conciliation (*n* = 3), transport difficulties (*n* = 2), and unwillingness to participate in group training (*n* = 1). 54 completed the final assessment.
Foulkes 2023 [19]	Usual Care	F = 50	51.2 ± 7.6	27.5 ± 5.6	12 discontinued the study. 6 at the 4-month follow-up due to health reasons (*n* = 3) and disinterest (*n* = 3). 6 at the 12-month follow-up due to COVID-19 concerns (*n* = 1), cannot contact (*n* = 3) and deceased (*n* = 2). 38 participants analyzed.
	Exercise	F = 52	50.3 ± 7.7	27.5 ± 4.6	4 discontinued the study. 3 at the 4-month follow-up due to health concerns. 1 at the 12-month follow-up due to time commitment issues. 49 participants analyzed.
Howden 2019 [20]	Usual Care	F = 14	52 ± 12	27.8 ± 6.2	N/A
	Exercise	F = 14	42 ± 9	25.2 ± 7.6	N/A
Kirkham 2020 [21]	Usual Care	F = 11	50 ± 10	26.7 ± 5.1	N/A
	Exercise	F = 26	49 ± 10	24.3 ± 4.5	1 stopped receiving anthracycline and 3 did not follow-up due to stress and treatment-related symptoms and were excluded from analyses. 3 participants completed the follow-up echocardiogram but not the VO_2_peak test due to treatment-related symptoms.
Naaktgeboren 2023 [10]	Usual Care	F = 72	58.5 ± 7.5	N/A	N/A
	Low Intensity ExT	F = 29	59.5 ± 10.2	N/A	N/A
	Moderate-High ExT	F = 80	59.1 ± 7.2	N/A	N/A
Kirkham 2023 [13]	Usual Care	F = 37	52 ± 9	27.8 ± 5.6	1 lost to follow-up.
	Exercise	F = 37	53 ± 10	27.4 ± 6.8	2 lost to follow-up.
Schneider 2023 [9]	EXduringAC	M = 2 F = 26	50 [38, 57]	23.0 [21.9, 26.4]	4 discontinued the study before the post-anthracycline visit. 3 were lost to follow-up.
	EXpostAC	M = 2 F = 27	46 [38, 57]	24.1 [21.5, 27.8]	2 discontinued the study before the post-anthracycline visit. 2 were lost to follow-up.

Age and BMI presented as mean ± STD. Age and BMI for Schneider 2023 is presented as median [1st and 3rd quartiles]

**Table 4:** Key findings from each study, comparing the exercise group(s) to the control group

Study ID	LVEF	GLS	VO_2_peak
Antunes 2023 [14]	☒	☒	☑↑
Diaz-Balboa 2024 [16]	☑*	☒	☑↑
Foulkes 2023 [19]	☑↑	☒	☑↑
Howden 2019 [20]	☒	☒	☑*
Kirkham 2020 [21]	☒	N/A	N/A
Naaktgeboren 2023 [10]	☒	☒	☒
Kirkham 2023 [13]	☒	☒	N/A
Schneider 2023 [9]	N/A	☒	☒

*Abbreviations*: ☒ non-significant difference, ☑ significant difference, * attenuated decline, ↑ improvement, *N/A* Not applicable, *GLS* Global longitudinal strain, *LVEF* Left ventricular ejection fraction

Antunes et al. conducted a randomized control trial (RCT) involving 93 females with early-stage BC scheduled to receive anthracycline-based chemotherapy, with 47 assigned to an AR-T intervention and 46 to usual care [21]. The 20–24-week intervention was delivered concurrently with treatment and included aerobic exercise (treadmill, cycling, or stepping) prescribed based on heart rate reserve (HRR), and progressive resistance training targeting both upper and lower body. Participants in the control group received no structured recommendations. The primary outcome was change in left ventricular ejection fraction (LVEF), with secondary outcomes including global longitudinal strain (GLS), LV volumes, LV mass index, left atrial volume index, stroke volume, cardiorespiratory fitness (VO_2_peak), cardiovascular biomarkers, and high-sensitivity cardiac troponin T. Immediately post-intervention, LVEF declined significantly in both groups: -1.6% (95% CI: -3.0, -0.1; *P* = 0.02) in the control group and − 0.8% (95% CI: -2.2, 0.5; *P* = 0.411) in the exercise group. At 3-months post intervention, LVEF declined further in both groups: -3.0% (95% CI: -4.5, -1.6, *P* < 0.001) in controls and − 1.9% (95% CI: -3.4, -0.57; *P* = 0.003) in the exercise group. Although the exercise group showed an attenuated decline at both timepoints, between-group differences were not statistically significant. No significant effects were observed for secondary cardiac outcomes, except for VO_2_ peak, which was significantly higher in the exercise group. The study concluded that AR-T during anthracycline treatment was safe, improved cardiorespiratory fitness, and did not exacerbate cardiotoxicity.

Diaz-Balboa et al. conducted a RCT to evaluate the effectiveness of a combined aerobic and resistance training (AR-T) program in preventing CTRCD in women with stage I-III BC [22, 23]. A total of 122 participants were randomized to either an exercise group (*n* = 60) or a control group receiving usual care with bi-monthly telephone based physical activity advice. The intervention lasted 3–12 months and consisted of twice-weekly, 60-minute AR-T sessions including resistance training with bodyweight and bands, and aerobic training at 50–85% HRR. Primary outcomes included changes in LVEF and GLS; secondary outcomes included cardiac biomarkers, VO_2_peak, and psychometric/lifestyle measures. Analysis of covariance (ANCOVA) showed a significant group difference in LVEF in favor of the exercise group post-intervention (*p* = 0.006, effect size = 0.4), with no change in GLS (*p* = 0.19). Per-protocol analysis showed a larger effect size for LVEF (0.7). Although LVEF declined in both groups, the decrease was significantly attenuated in the intervention group [-1.5% (95% CI: -2.9, -0.1); *p* = 0.006]. No significant changes were observed in GLS or biomarkers. No cases of CTRCD occurred. The authors concluded that AR-T is a safe adjunct therapy that may attenuate LVEF decline during anthracycline and/or HER2-targeted treatment.

Foulkes et al. conducted a RCT to investigate the effects of a 12-month combined AR-T program on female BC patients scheduled for anthracycline-based chemotherapy [24, 25]. A total of 104 patients were randomized with 52 in the intervention group. The AR-T program included moderate-intensity endurance, tempo, and high-intensity interval training, along with moderate-to-high intensity resistance training. Exercise intensity was progressively increased weekly, with a deloading week following each chemotherapy cycle. The primary outcome examined was VO_2_peak, while secondary outcomes included cardiac output (CO), stroke volume (SV), LVEF, and right ventricular ejection fraction (RVEF). VO_2_peak declined in both groups at 4 months, but the decrease was attenuated in the AR-T group (6% vs. 13% reduction), resulting in a significant between-group difference (1.5 ml/kg/min; *P* = 0.003). At 12 months, VO_2_peak improved by 9% in the exercise group and remained 7% below baseline in controls (*P* = 0.001). Exercise-induced increases in CO, SV, LVEF, and RVEF were significantly greater in the intervention group at 4 and 12 months (*P* < 0.001). No significant differences were found in resting LVEF, GLS, or diastolic function. Few participants in either group met cardiotoxicity criteria. The authors concluded that AR-T is a safe and effective adjunct therapy that may preserve or improve cardiac function during chemotherapy.

Howden et al. conducted a non-RCT involving 28 BC patients scheduled to receive anthracycline-based chemotherapy [26]. The study aimed to assess whether a combined AR-T intervention could attenuate declines in VO_2_peak and whether exercise cardiac imaging had superior sensitivity for detecting cardiac injury compared to standard resting LVEF measures. Participants were assigned to either an AR-T group or usual care. The intervention began after the first anthracycline dose and included two weekly 60-minute supervised sessions and one home-based 30–60-minute aerobic session. Programs were individualized and progressively intensified. Primary outcomes included VO_2_peak, resting ECG, LVEF, GLS, and cardiac biomarkers, while secondary outcomes examined were SV and peak CO. Both groups experienced declines in VO_2_peak, though the reduction was significantly attenuated in the exercise group (4% vs. 15%; *P* = 0.010). Fewer participants in the AR-T group met the criteria for functional disability (VO_2_peak < 18 mL/kg/min). Post-treatment, resting LVEF decreased, and troponin levels increased significantly in both groups, with no between-group differences. Peak exercise CO was identified as the strongest predictor of functional capacity post-anthracycline therapy. The authors concluded that AR-T may prevent chemotherapy-related functional disability, though results should be interpreted cautiously due to the non-randomized design and potential for exercise in the control group.

Kirkham et al. conducted a prospective non-randomized controlled study of 37 BC patients scheduled to receive anthracycline-based chemotherapy to examine the effects of an AR-T program on resting cardiac function and hemodynamics [27]. The intervention involved three weekly sessions of aerobic exercise (treadmill, elliptical, or cycle ergometer, 20–30 min at 50–75% HRR) and whole-body resistance training at moderate intensity. Usual care participants received no formal intervention. The primary outcome examined was GLS, with secondary outcomes including CO, SV, BP, and systemic vascular resistance (SVR). Assessments occurred within two weeks prior to and after chemotherapy treatment. VO_2_peak was also measured. No significant differences were observed in GLS, ventricular volumes, ejection fraction, or diastolic function between groups. Hemoglobin, hematocrit, and mean arterial pressure decreased in both the exercise and the usual care groups (*p <* 0.05). Resting CO increased only in the control group (+ 0.27 ± 0.24 L/min/m2; *p* = 0.03), which was attributed to elevated heart rate. SVR decreased in both groups but was significantly attenuated in the exercise group (*p* = 0.03), despite similar hematocrit reductions. Reduced vessel lumen radius was found in the exercise group (*p* = 0.08), with Kirkham and colleagues noting this was due to a compensatory mechanism mitigating SVR decline. The authors concluded that while AR-T did not preserve resting cardiac function, it did appear to modify vascular responses, potentially counteracting chemotherapy-induced hemodynamic changes.

Naaktgeboren et al. conducted an 8-year follow up of BC survivors from two RCTs (PACT and PACES; 2009–2013) [10–12]. 185 participants with a mean age of 58.9 years who were previously treated with anthracyclines (PACT) or trastuzumab (PACES) were included in the cohort. Participants in the intervention groups of these respective studies performed AR-T. Further details of the adopted exercise protocols can be found in Table 2. Cardiotoxicity was assessed via multimodality imaging, including measures of cardiac magnetic resonance (CMR)-derived extracellular volume fraction (ECV) expressed as a percentage of the extracellular myocardial volume, LVEF, and GLS. VO_2_ peak and physical activity (PA) levels were also measured. Mean ECV was within normal ranges across groups (25.3% ± 2.5%), 24.6% ± 2.8% in the moderate to high intensity exercise group, and 25.5% ± 2.7% in the low intensity exercise group. Borderline normal mean LVEF values were found in all 3 groups, with the lowest mean LVEF in the moderate to high intensity exercise group (53.0 ± 7.8%); this group also had the largest proportion of participants with an LVEF meeting the criteria of cardiotoxicity (LVEF < 50%), with 22 of the 79 in the group. GLS was borderline normal in all groups, but abnormal values were common. Diastolic function was reported to be normal in half the participants with most diastolic dysfunction reported in the moderate to high intensity group. Moderate to high intensity exercise was found to not have a significant effect on ECV (β=−0.69, 95% CI: −1.62 to 0.25). No benefit of exercise was found for LVEF or GLS (OR 1.67, 95% CI 0.72 to 3.99); OR 1.34, 95% CI 0.63 to 2.88), respectively). Mean native T1 was normal in the moderate to high intensity exercise group but elevated in the control group. It was concluded that in terms of functional cardiac parameters measured, specifically LVEF and GLS, there was a non-significant less favorable outcome in the exercise groups.

The TITAN study was a RCT conducted by Kirkham et al. for 74 BC patients (≥ 18 years), testing a comprehensive cardiac rehabilitation care model versus usual care [13]. The intervention included twice-weekly, 60–90 min moderate intensity combined AR-T, individualized progression, optional home exercise, cardiovascular risk management, and dietary counselling. The primary outcome examined was cardiac MRI-derived LVEF. Secondary outcomes measured included cardiotoxicity (LVEF drop >10% to < 53%), GLS, cardiac biomarkers (brain natriuretic peptide (BNP), high-sensitivity troponin-1), cardiovascular risk factors (PA, blood pressure, lipid profile, glucose), and physical fitness (VO_2_peak, muscular strength, and body composition). Assessments of outcomes were conducted at baseline, 24-weeks post chemotherapy, and 52 weeks, excluding body composition, blood pressure, and biomarkers, which were not measured at 24 weeks. No between-group differences were found for LVEF, cardiotoxicity incidence, GLS, or BNP. The only significant difference found was reduced total cholesterol (5.2 ± 0.8 mmol/L to 4.7 ± 0.8 mmol/L) in the intervention group. Authors concluded that the low cardiotoxicity incidence, absence of early treatment-period assessments (6–12 weeks), lack of a long-term follow-up (post 52-weeks), and limited statistical power likely reduced the ability to detect differences between groups.

A RCT study carried out by Schneider et al. examined the effect of supervised AR-T in 57 adults (BC or lymphoma; 4 males) scheduled for anthracycline treatment [9]. Patients were randomized into supervised AR-T either concurrent with treatment or post-treatment. Exercise consisted of 30 min of cycling with intensity based on patient ventilatory threshold, followed by 40–45 min of strength training (70–80% of patient’s 1-repetition-max, 2–3 sets of 8–12 repetitions) for all muscle groups (targeting rating of perceived exertion of 15) coordination, and/or balance training. 24 supervised sessions and 12 non-supervised home-based sessions were prescribed. The intervention lasted 12 weeks for the control group (exercise post-anthracycline) and there was no strict duration for the concurrent training group due to individual anthracycline treatment lengths. The primary outcome examined was GLS pre- and post-anthracycline. Secondary outcomes measured included high sensitivity troponin-T (hsTnT), NT-pro-brain natriuretic peptide (NT-proBNP), VO_2_peak, and objectively measured PA. Both groups showed non-significant GLS declines (7.4% concurrent; 1.0% post) with no between-group differences. Changes did not exceed the clinical threshold of 15% associated with future reductions in ejection fraction. Greater PA was associated with more favorable biomarker profiles – 20 extra daily steps predicted 1% more negative GLS, and 30 more minutes of moderate-to-vigorous activity (MVPA) predicted 1.8% more negative GLS and 3 ng/L lesser increase in hsTnT. VO_2_peak decreased in both groups. The authors concluded that supervised exercise was not superior to PA tracking alone in mitigating cardiotoxicity, however maintaining higher PA during chemotherapy was linked to cardioprotective biomarker patterns.

## Discussion

The current review highlights the lack of evidence behind the perceived hesitation to prescribe resistance training for cancer survivors and patients who have received anthracyclines. While aerobic exercise is often prescribed to combat the cardiotoxic effects of anthracycline treatment in patients, little is known about the potential effects of resistance training on anthracycline-induced cardiotoxicities [22, 23]. The examined literature evaluated the effect of resistance training on cardiac function in cancer patients who were receiving or who had received anthracyclines. This systematic review highlights (1) the need for more research on the effect of resistance training per se on pediatric and adult cancer survivors who have received anthracycline treatment; (2) the complex and nuanced effects of AR-T as an adjunct therapy for BC patients undergoing anthracycline treatment; and (3) the fact that there is no evidence that demonstrates a negative impact of resistance training on patients treated with anthracyclines.

Historically, in the absence of definitive exercise guidelines for cancer survivors treated with cardiotoxic therapies, resistance training was often approached cautiously [24]. These concerns were primarily directed toward survivors who exhibited reduced left ventricle wall thickness or left ventricle mass and therefore had a potentially higher susceptibility to increases in afterload. It was hypothesized that the transient elevations in intrathoracic and arterial pressures during resistance exercise, particularly when associated with the Valsalva maneuver, might impose excessive wall stress on an anthracycline-weakened myocardium [25–28]. Based on the results of this review, there is no evidence that resistance training negatively impacts cardiac structure or function in patients treated with anthracyclines.

While it was theorized that resistance training would improve or prevent anthracycline-induced cardiac dysfunction, only Foulkes et al., and Diaz-Balboa et al. found significant positive differences in cardiac function, specifically LVEF, between the AR-T and control groups [16, 19]. Furthermore, only Foulkes et al. found a significant improvement in LVEF during AR-T, while Diaz-Balboa et al. only found an attenuated drop in LVEF after anthracycline treatment in the AR-T group [16, 19]. No other included study found a statistically significant effect of combined AR-T on LVEF or GLS in patients who had received anthracycline treatment. In fact, one follow-up study performed after an average of 8.5 years post-exercise intervention found less favourable results for LVEF and GLS in the AR-T groups compared to the control group. The authors emphasized, however, that this should not be interpreted to indicate a negative effect of AR-T on cardiac functioning since there was no baseline LVEF or GLS for comparison. Rather, the authors encourage the conduct of additional high-quality prospective RCTs to confirm or refute this finding [10]. While there are currently conflicting results on the effect of AR-T on cardiac function in patients who have received anthracycline treatment, no studies have reported a statistically significant negative effect of combined AR-T.

Importantly, these findings align with broader evidence supporting exercise as foundational to survivorship care. A study conducted by Rock et al. showed that postdiagnosis physical activity improves cancer-specific and all-cause mortality, informing current American Cancer Society guidelines recommending 150–300 min of moderate-to-vigorous physical activity weekly for adults and at least 60 min daily for children and adolescents [29]. Furthermore, recent findings by Mayr et al. show that higher cardiopulmonary fitness is associated with a reduced burden of chronic adverse health outcomes, particularly musculoskeletal and cardiovascular disorders, highlighting the importance of improving both aerobic capacity and muscular strength in cancer survivors [30]. Our review supports these conclusions, as most studies examined demonstrated improved VO_2_peak and either preserved or improved LVEF with exercise consisting of both aerobic and resistance training (Table 4). Findings from the St. Jude Lifetime Cohort study also demonstrated that cancer survivors who were previously exposed to anthracycline or chest radiation chemotherapy and achieved higher cardiopulmonary fitness levels, had significantly lower risk of serious CVD compared to their inactive peers [31]. As all 3 studies reviewed the use of AR-T, this evidence collectively reinforces the idea that resistance exercise is not only safe, but may be an essential component of exercise prescription for individuals treated with anthracycline, as our review identified no evidence of resistance-exercise induced decline in cardiac function, and the observed improvements shown in Table 4 show a mechanism by which exercise can reduce mortality and chronic disease progression in this population.

Our study cannot ascertain the effects of resistance training alone on anthracycline-induced cardiotoxicity due to the inclusion of both aerobic and resistance training in the interventions. We also cannot make conclusions about the pediatric population as there is currently no literature that has explored the effects of resistance training on pediatric patients who have received anthracycline treatment. Nevertheless, our investigation provides useful information for clinicians who discourage resistance training for patients who have received anthracycline treatment, as none of the studies support the theory that resistance training can be harmful to patients treated with anthracyclines. Therefore, future research should focus on conducting more high-quality clinical RCTs, specifically with one arm of the exercise intervention including resistance training only, to isolate the effects of resistance training and remove the stigma surrounding its prescription for patients treated with anthracyclines in the adult and pediatric populations.

### Limitations

One limitation to this systematic review was that the studies included only featured combined AR-T, therefore any statistically significant differences between groups or lack thereof cannot be solely attributed to resistance training alone. As there were no studies that featured solely resistance training and measures of cardiac function (i.e., LVEF and GLS), the authors opted to include the combined AR-T to highlight the limited evidence to suggest that resistance training can negatively impact patients treated with anthracyclines.

Secondly, although all studies had combined AR-T as their intervention, all studies had different parameters for frequency, intensity, time, type, and duration of the exercise intervention. Hence, the results of this review cannot be considered conclusive evidence for the effects of resistance training on cardiac function in patients treated with anthracyclines.

Additionally, each study was performed with only female participants aged 18 or older, thus any findings may not be applicable to childhood and adolescent cancer survivors. Furthermore, only one study included four male participants, and all other studies had only female BC participants, which could bias the findings and reduce the generalizability of the results for sex-related differences.

## Conclusion

Without definitive evidence, the belief that resistance training can negatively impact patients with anthracycline-induced cardiac dysfunction is unsupported. While no conclusion can be made regarding the effect of resistance training on cardiac function, resistance training has been shown to improve cardiorespiratory fitness, which can be directly related to improved quality of life. This review demonstrates a need for high-quality clinical research to be conducted to ascertain the isolated effects of resistance training on patients experiencing anthracycline-induced cardiotoxicity, for possible inclusion into exercise guidelines for adult and pediatric populations.

## Data Availability

Datasets are available from the corresponding author upon reasonable requests.

## Abbreviations

AR-T: Aerobic and resistance training
BC: Breast cancer
CO: Cardiac output
CTRCD: Cancer therapy-related cardiac dysfunction
CVD: Cardiovascular disease
GLS: Global longitudinal strain
LVEF: Left ventricular ejection fraction
SV: Stroke volume
VO_2_peak: Peak oxygen uptake

## References

[1] Siegel RL, Giaquinto AN, Jemal A. Cancer statistics, 2024. CA Cancer J Clin. 2024;74(1):12–49. 38230766 10.3322/caac.21820

[2] Lyon AR, López-Fernández T, Couch LS, Asteggiano R, Aznar MC, Bergler-Klein J, et al. 2022 ESC guidelines on cardio-oncology developed in collaboration with the European hematology association (EHA), the European society for therapeutic radiology and oncology (ESTRO) and the international Cardio-Oncology society (IC-OS). Eur Heart J. 2022;43(41):4229–361. 36017568 10.1093/eurheartj/ehac244

[3] Camilli M, Cipolla CM, Dent S, Minotti G, Cardinale DM. Anthracycline cardiotoxicity in adult cancer patients. JACC: CardioOncology. 2024;6(5):655–77. 39479333 10.1016/j.jaccao.2024.07.016 PMC11520218

[4] Plana JC, Galderisi M, Barac A, Ewer MS, Ky B, Scherrer-Crosbie M, et al. Expert consensus for multimodality imaging evaluation of adult patients during and after cancer therapy: a report from the American society of echocardiography and the European association of cardiovascular imaging. Eur Heart J - Cardiovasc Imaging. 2014;15(10):1063–93. 25239940 10.1093/ehjci/jeu192 PMC4402366

[5] Rahman AM, Yusuf SW, Ewer MS. Anthracycline-induced cardiotoxicity and the cardiac-sparing effect of liposomal formulation. Int J Nanomed. 2007;2(4):567–83. PMC2676818 18203425

[6] Bates JE, Howell RM, Liu Q, Yasui Y, Mulrooney DA, Dhakal S, et al. Therapy-related cardiac risk in childhood cancer survivors: an analysis of the childhood cancer survivor study. JCO. 2019;37(13):1090–101. 10.1200/JCO.18.01764 PMC6494356 30860946

[7] Ryan TD, Bates JE, Kinahan KE, Leger KJ, Mulrooney DA, Narayan HK et al. Cardiovascular Toxicity in Patients Treated for Childhood Cancer: A Scientific Statement From the American Heart Association. Circulation. 2025;151(15). Available from: https://www.ahajournals.org/10.1161/CIR.0000000000001308. Cited 2025 Apr 25. 10.1161/CIR.0000000000001308 PMC12265934 40104841

[8] Venkatesh P, Kasi A. Anthracyclines. In: StatPearls. Treasure Island (FL): StatPearls Publishing; 2025. Available from: http://www.ncbi.nlm.nih.gov/books/NBK538187/. Cited 2025 July 14.

[9] Schneider C, Ryffel C, Stütz L, Rabaglio M, Suter TM, Campbell KL, et al. Supervised exercise training in patients with cancer during anthracycline-based chemotherapy to mitigate cardiotoxicity: a randomized-controlled-trial. Front Cardiovasc Med. 2023;10:1283153. 38111886 10.3389/fcvm.2023.1283153 PMC10725952

[10] Naaktgeboren WR, Stuiver MM, Van Harten WH, Aaronson NK, Scott JM, Sonke G, et al. Effects of exercise during chemotherapy for breast cancer on long-term cardiovascular toxicity. Open Heart. 2023;10(2):e002464. 37903570 10.1136/openhrt-2023-002464 PMC10619040

[11] Travier N, Velthuis MJ, Steins Bisschop CN, Van Den Buijs B, Monninkhof EM, Backx F, et al. Effects of an 18-week exercise programme started early during breast cancer treatment: a randomised controlled trial. BMC Med. 2015;13(1):121. 26050790 10.1186/s12916-015-0362-z PMC4461906

[12] Van Waart H, Stuiver MM, Van Harten WH, Geleijn E, Kieffer JM, Buffart LM, et al. Effect of Low-Intensity physical activity and Moderate- to High-Intensity physical exercise during adjuvant chemotherapy on physical Fitness, Fatigue, and chemotherapy completion rates: results of the PACES randomized clinical trial. JCO. 2015;10(17):1918–27. 10.1200/JCO.2014.59.1081 25918291

[13] Kirkham AA, Mackey JR, Thompson RB, Haykowsky MJ, Oudit GY, McNeely M, et al. TITAN Trial JACC: Adv. 2023;2(6):100424. 38939428 10.1016/j.jacadv.2023.100424 PMC11198667

[14] Antunes P, Joaquim A, Sampaio F, Nunes C, Ascensão A, Vilela E, et al. Effects of exercise training on cardiac toxicity markers in women with breast cancer undergoing chemotherapy with anthracyclines: a randomized controlled trial. European Journal of Preventive Cardiology. 2023;30(9):844–55. 36857149 10.1093/eurjpc/zwad063

[15] Díaz-Balboa E, González-Salvado V, Rodríguez-Romero B, Martínez-Monzonís A, Pedreira-Pérez M, Palacios-Ozores P, et al. A randomized trial to evaluate the impact of exercise-based cardiac rehabilitation for the prevention of chemotherapy-induced cardiotoxicity in patients with breast cancer: ONCORE study protocol. BMC Cardiovasc Disord. 2021;21(1):165. 33827450 10.1186/s12872-021-01970-2 PMC8025895

[16] Díaz-Balboa E, Peña-Gil C, Rodríguez-Romero B, Cuesta-Vargas AI, Lado-Baleato O, Martínez-Monzonís A, et al. Exercise-based cardio-oncology rehabilitation for cardiotoxicity prevention during breast cancer chemotherapy: the ONCORE randomized controlled trial. Prog Cardiovasc Dis. 2024;85:74–81. 38395212 10.1016/j.pcad.2024.02.002

[17] Foulkes SJ, Howden EJ, Antill Y, Loi S, Salim A, Haykowsky MJ, et al. Exercise as a diagnostic and therapeutic tool for preventing cardiovascular morbidity in breast cancer patients– the breast cancer exercise intervention (BREXIT) trial protocol. BMC Cancer. 2020;20(1):655. 32664946 10.1186/s12885-020-07123-6 PMC7362469

[18] Giantris A, Abdurrahman L, Hinkle A, Asselin B, Lipshultz SE. Anthracycline-induced cardiotoxicity in children and young adults. Crit Rev Oncol/Hematol. 1998;27(1):53–68. 9548017 10.1016/s1040-8428(97)10007-5

[19] Foulkes SJ, Howden EJ, Haykowsky MJ, Antill Y, Salim A, Nightingale SS, et al. Exercise for the prevention of Anthracycline-Induced functional disability and cardiac dysfunction: the BREXIT study. Circulation. 2023;147(7):532–45. 36342348 10.1161/CIRCULATIONAHA.122.062814

[20] Howden EJ, Bigaran A, Beaudry R, Fraser S, Selig S, Foulkes S, et al. Exercise as a diagnostic and therapeutic tool for the prevention of cardiovascular dysfunction in breast cancer patients. Eur J Prev Cardiolog. 2019;26(3):305–15. 10.1177/2047487318811181 30376366

[21] Kirkham AA, Virani SA, Bland KA, McKenzie DC, Gelmon KA, Warburton DER, et al. Exercise training affects hemodynamics not cardiac function during anthracycline-based chemotherapy. Breast Cancer Res Treat. 2020;184(1):75–85. 32816189 10.1007/s10549-020-05824-x

[22] Kang DW, Wilson RL, Christopher CN, Normann AJ, Barnes O, Lesansee JD, et al. Exercise Cardio-Oncology: exercise as a potential therapeutic modality in the management of Anthracycline-Induced cardiotoxicity. Front Cardiovasc Med. 2022;8:805735. 35097024 10.3389/fcvm.2021.805735 PMC8796963

[23] Kouzi SA, Uddin MN. Aerobic exercise training as a potential cardioprotective strategy to attenuate Doxorubicin-Induced cardiotoxicity. J Pharm Pharm Sci. 2016;19(3):399. 27806245 10.18433/J3JS5R

[24] Okada M, Meeske KA, Menteer J, Freyer DR. J Pediatr Oncol Nurs. 2012;29(5):246–52. 22907680 10.1177/1043454212451525

[25] Landier W, Bhatia S, Eshelman DA, Forte KJ, Sweeney T, Hester AL, et al. Development of Risk-Based guidelines for pediatric cancer survivors: the children’s oncology group Long-Term Follow-Up guidelines from the children’s oncology group late effects committee and nursing discipline. JCO. 2004;22(24):4979–90. 10.1200/JCO.2004.11.032 15576413

[26] Philip LJ, Findlay SG, Gill JH. Baseline blood pressure and development of cardiotoxicity in patients treated with anthracyclines: A systematic review. Int J Cardiol Cardiovasc Risk Prev. 2022;15:200153. 36573186 10.1016/j.ijcrp.2022.200153 PMC9789356

[27] Smith WA, Ness KK, Joshi V, Hudson MM, Robison LL, Green DM. Exercise training in childhood cancer survivors with subclinical cardiomyopathy who were treated with anthracyclines. Pediatr Blood Cancer. 2014;61(5):942–5. 10.1002/pbc.24850 PMC4167576 24623535

[28] Stricker PR, Faigenbaum AD, McCambridge TM, COUNCIL ON SPORTS MEDICINE AND FITNESS, LaBella CR, Brooks MA et al. Resistance Training for Children and Adolescents. Pediatrics. 2020;145(6):e20201011. 10.1542/peds.2020-1011 32457216

[29] Rock CL, Thomson CA, Sullivan KR, Howe CL, Kushi LH, Caan BJ, et al. American cancer society nutrition and physical activity guideline for cancer survivors. CA Cancer J Clin. 2022;72(3):230–62. 35294043 10.3322/caac.21719

[30] Mayr AK, Zürcher S, Bänteli I, Hebestreit H, Kasteler R, Von Der Weid NX, et al. Physical fitness and clinically assessed disease burden in long-term childhood cancer survivors—The SURfit study. Cancer. 2025;131(17):e70051. 40831029 10.1002/cncr.70051 PMC12365372

[31] Wogksch MD, Ware ME, Onerup A, O’Neil ST, Nolan VG, Smeltzer MP et al. Associations between Cardiopulmonary Fitness and Cardiovascular Events in Survivors of Childhood Cancer: A Report from the St. Jude Lifetime Cohort. Medicine & Science in Sports & Exercise. 2025; Available from: https://journals.lww.com/10.1249/MSS.0000000000003802. Cited 2025 Oct 25. 10.1249/MSS.0000000000003802 PMC12379817 40590682

